# A Task- and Role-Oriented Design Method for Multi-User Collaborative Interfaces

**DOI:** 10.3390/s25061760

**Published:** 2025-03-12

**Authors:** Xiaoxi Du, Menglian Yu, Zichen Zhang, Mu Tong, Yanfei Zhu, Chengqi Xue

**Affiliations:** 1 School of Mechanical Engineering, Southeast University, Nanjing 211189, China; duxiaoxi@seu.edu.cn (X.D.); 220214991@seu.edu.cn (M.Y.); zzc118226@126.com (Z.Z.); zhuyf@seu.edu.cn (Y.Z.); 2 Nanjing Vivo Software Technology Co., Ltd., Nanjing 210012, China; 3 School of Design, South China University of Technology, Guangzhou 510641, China; tongmu@scut.edu.cn

**Keywords:** collaborative interface, multi-user, task, role, design method

## Abstract

In multi-user collaborative interaction systems, the interface serves not only as a medium for human–computer interaction but also as a crucial channel for communication between users. Consequently, the quality of collaborative interface design directly impacts the overall effectiveness of the system. In collaborative systems, different users typically assume distinct roles, and task flows are typically more complex. Compared to single-user interfaces, multi-user collaborative interfaces must account for a broader range of collaboration requirements and characteristics. Although a substantial body of theoretical and practical research on user interface design exists, design methods specifically for multi-user collaborative interaction interfaces are still lacking. Therefore, this study builds on the existing theories and case studies of collaborative systems, extending user-centered design methods. The study emphasizes the analysis of task flows and role relationships in multi-user collaboration and integrates collaboration needs and characteristics throughout every stage of the interface design process. Ultimately, we propose a methodological framework for interface design tailored to multi-user collaborative interaction systems, aiming to provide theoretical support for the development of more advanced and comprehensive collaborative systems.

## 1. Introduction

The development of globalization and advancements in information and interaction technologies have increasingly characterized human activities by their collective, interactive, distributed, and cooperative nature [1]. Collaboration, defined as a joint and interdependent activity to achieve common goals [2], has become central to modern organizational work structures. Within this context, Computer-Supported Cooperative Work (CSCW) systems [3,4,5] meet the demands of collaborative activities by providing communication, coordination, and information-sharing functionalities.

The COVID-19 pandemic has accelerated changes in work habits [6], significantly increasing the use of collaborative applications and tools across various environments [7]. Multi-user collaboration across time and space is now the norm. In software development, developers use GitHub for code collaboration and version control. In education and training, collaborative learning is believed to promote knowledge construction and higher-order thinking skills [8,9], with institutions utilizing platforms like Google Classroom and Microsoft Teams to support online teaching and student cooperation. In business management, tools such as Trello and Asana help teams plan projects and track progress. In design, Figma and Axure enable real-time idea sharing and feedback. These tools have greatly enhanced collaboration efficiency.

Most modern applications are built using window managers, toolkits, and interface builders [10]. The interface, as the carrier of information and functions, is the only part of the system that users can see, hear, or touch. For users, the interface is the system [11]. This is particularly true in collaborative systems involving multi-user collaboration technologies and complex interactions between users. Interfaces serve not only as the medium for human–computer interaction but also as channels for communication between people. The quality of collaborative interfaces is crucial to the entire collaborative system, directly affecting the smoothness of team communication and the effectiveness of task coordination, thus impacting overall team collaboration quality. Therefore, designing multi-user collaborative interfaces requires considering not only single-user intentions and behaviors but also multi-user needs, constraints, and expectations [12].

In multi-user collaborative systems, tasks are often complex, involving multiple levels and steps, with each part potentially requiring specific expertise and skills. Thus, collaborative tasks necessitate team members with different experiences and knowledge backgrounds to take on specific roles [13] and responsibilities, working together to achieve the desired collective outcome [14]. Roles represent the “who” and tasks represent the “what”; combining them provides a clear understanding of “who is doing what” in the collaboration process. Consequently, compared to single-user interfaces, focusing on task flow and role relationships [15] is particularly important in the design of collaborative interfaces.

Collaborative interface design requires guidelines to ensure that the designed interfaces are rational and meet different and complex collaboration needs. Although there is extensive theoretical and practical research on user interface design [16,17,18], studies specifically focusing on multi-user collaborative interface design methods are still lacking. Most related research delves into collaborative task analysis [19], collaboration model construction [20,21], and designing specific solutions for collaborative issues. However, there is a lack of a systematic methodology to guide the entire process of collaborative interface design.

Therefore, this study aims to integrate multi-user collaboration considerations into all stages of the interface design process, with a particular emphasis on the analysis of task flows and role relationships. The goal is to develop a comprehensive interface design methodology that spans the entire design process, assisting designers and developers of collaborative systems in creating and optimizing interfaces that enhance collaborative interaction features and meet the multi-user interaction requirements across various collaborative contexts. Furthermore, this study reviews and synthesizes existing perspectives and solutions related to multi-user collaboration systems, incorporating them into the proposed method. These research findings not only serve as the foundational basis for the proposed method but also provide both theoretical and practical references for a deeper understanding of the approach.

The structure of this paper is organized as follows. Section 2 introduces the foundational research relevant to this study. Section 3, Section 4, Section 5, Section 6 and Section 7 outline our design methodology framework and provide detailed explanations of each key component. In Section 8, we discuss the limitations of our design method and explore potential future directions. Furthermore, Section 9 concludes the paper with a summary of the key findings.

## 2. Related Works

This section introduces the theoretical foundation related to this study, focusing on collaboration models and construction methods, as well as collaborative interfaces and design methods.

### 2.1. Collaboration Models and Construction Methods

Collaboration models describe and analyze collaborative relationships, providing insight into team dynamics and workflow structures, thereby forming a theoretical foundation for computer applications known as groupware. These models categorize collaboration along two dimensions, as defined by Johansen’s matrix: time (synchronous and asynchronous) and space (co-located and distributed) [22]. Within these dimensions, four main collaborative work models emerge: the Conversation Model, which views the process as a series of conversational activities; the Meeting Model, which focuses on group members sharing a workspace to discuss and make decisions; the Process Model, which emphasizes the logic and sequence of tasks and operations; and the Activity Model, based on activity theory, which provides a comprehensive view that includes task decomposition, division of labor, task goals, and relationships [23,24,25,26]. Together, these models offer a structured approach to understanding and facilitating effective collaboration.

In team collaboration, members often have different skills, priorities, knowledge, or experience, leading to different roles [13,27]. Roles are foundational concepts in constructing collaborative systems [28], serving as abstract descriptions of individual behaviors in collaborative work. Roles are critical in the design of information systems [29], and they help designers and developers understand collaborative relationships and processes. Drury and Williams (2002) proposed a role-based awareness support specification and evaluation framework for analyzing users’ awareness needs in synchronous collaboration applications and evaluating awareness-related functions [30]. Zhu and Zhou (2006) introduced the E-CARGO role-based collaboration model and built a role-based collaborative system architecture on this model [31]. They also proposed a role-based software engineering process and developed prototype tools for dynamically adding, assigning, and modifying roles [32]. Building on this, Zhu (2021) further presented a more systematic computational method using roles as a key mechanism to facilitate collaborative activities, helping researchers and practitioners address complex issues in collaborative systems and technologies [33]. With advancements in artificial intelligence, research on collaboration models has begun incorporating intelligent agents as participants, introducing the concept of role agents to facilitate human–computer collaboration [34]. Studies have shown that through roles and role agents, human users can better understand how to use collaborative systems and achieve higher collaborative productivity [35].

Role-based construction methods bind user permissions to roles, describing and defining the static aspects of group collaboration. This approach often necessitates frequent role changes to adapt to the evolving context of tasks, which overlooks the dynamic nature of collaborative relationships. Basu and Blanning (2000) suggested that when modeling collaborative processes, it is important to focus on information, tasks, and resources, specifically clarifying the types of information elements for computing other elements, analyzing task dependencies, and understanding how resource availability impacts information and tasks [36]. Increasingly, scholars analyzing collaborative processes consider not only collaborative roles but also tasks and other factors. Wurdel, Sinnig, and Forbrig (2009) proposed a task-based normative framework to model participants, roles, and collaborative tasks in a collaborative environment [37]. Patel, Pettitt, and Wilson (2012) summarized context, support, tasks, interaction processes, teams, individuals, and overarching factors into a foundational framework, developing a collaborative work model named CoSpaces [19]. Madani, Erradi, and Benkaouz (2016) proposed a Collaboration Task Role-Based Access Control (CTRBAC) model to ensure access control over shared resources in collaborative sessions within multi-user environments [38].

In summary, combining the concepts of roles and tasks provides a clear depiction of collaborative relationships within teams and helps manage complex collaborative processes.

### 2.2. Collaborative Interfaces and Design Methods

Collaborative interfaces play a crucial role in modern information systems and work environments. Utilizing new technologies to design and develop collaborative interfaces has proven effective in addressing various collaboration issues, enhancing efficiency, and improving user experience. For example, Du et al. (2022) used Kinect devices for multi-user identification and real-time motion tracking, designing personalized shortcut menus to support multi-user collaboration on large interactive display tables [39]. Chen et al. (2024) found that exocentric perspective interfaces in VR environments significantly improved task performance, usability, social presence, and user experience in remote collaboration tasks while reducing VR motion sickness [40]. Jing et al. (2022) reported a 360° panoramic mixed reality (MR) remote collaboration system that visualized gaze behavior sharing between local users in augmented reality and remote collaborators in virtual reality, enhancing shared attention and co-presence [41]. Matsuda and Komuro (2020) proposed an interface allowing multiple users to interactively access information and operate simultaneously on large displays, optimizing screen layout dynamically to prevent interference between users [42]. Kirkbride (2020) developed novel collaborative interfaces to help musicians collaboratively code computer-generated music live in front of audiences, exploring the impact of user interface design parameters on group creativity [43]. Ivanyi et al. (2023) introduced a Collaborative Accessible Digital Music Interface (CADMI) based on eye-tracking and head and mouse interactions, providing remote collaborative real-time music creation for people with amyotrophic lateral sclerosis (PALS) [44]. Neogy, Zong, and Satyanarayan (2020) developed a design space for multiple users to collaborate on visualizations, introducing techniques to track collaborators’ activities and allow users to follow their own paths [45]. These studies provide rich cases and ideas for optimizing collaborative interface design. Additionally, several studies have proven the importance of collaboration and interaction among project team members in user interface design [46,47]. For example, Sangiorgi, Beuvens, and Vanderdonckt (2012) introduced the Gambit multi-platform system, allowing multiple users to draw sketches using HTML5 to design graphical user interface prototypes, aiding collaborative interface design [48].

Numerous principles and guidelines ensure that interface design is rational and systematic. Popular design methods include the product development life cycle (PDLC) [49], double diamond [50,51], participatory design (PD) [52], iterative design [53,54], and user-centered design (UCD) [55]. These methods typically emphasize an iterative, cyclical design process, driven by user research from problem definition to solution development. Among these, user-centered design (UCD) is widely popular and has become a crucial concept in interactive system design. It involves designing socio-technical systems considering not only users but also the application of technology in users’ daily activities [56]. The ISO 13407 standard [57] outlines a user-centered design process involving four activities: (1) understanding and specifying the context of use, (2) specifying the user and organizational requirements, (3) producing design solutions, and (4) evaluating designs against requirements. However, there is no consensus on the methods to be used at each stage of the UCD framework [58]. Moreover, although methods like UCD cover the process from considering user opinions in software product development to evaluating the outcomes, they focus more on single-user design, neglecting the impact of other users’ intentions and behaviors in one group. Recently, scholars have tried incorporating multi-user considerations into design methods. For instance, Fleury and Chaniaud (2024) proposed multi-user experience (MUX) guidelines, the multi-user acceptance model (MAM), and multi-user centered design (MCD) [12]. Carcani et al. (2023) improved the participatory design method to address power issues in collaborative design, enabling equal collaboration among participants [59].

In summary, existing research has proposed various interface design solutions to improve collaboration efficiency and user experience, and these solutions often focus on leveraging new technologies to address specific issues in particular collaboration scenarios. Additionally, while many classic design methods provide guidance for interface design, most are centered on individual users. These research outcomes not only establish a solid foundation for advancing studies on multi-user collaborative systems but also demonstrate the potential value of developing a comprehensive and generalizable interface design methodology specifically tailored to multi-user collaborative environments.

Through the above analysis and summary, we find that the concepts of roles and tasks are fundamental to the analysis and design of multi-user collaborative systems, particularly in complex environments where multiple users interact and collaborate through technological platforms. These systems include, but are not limited to, virtual collaboration environments, remote team platforms, and group decision support systems. Furthermore, the core value of the user-centered design methodology lies in its well-established normative and systematic principles, as well as its capacity to provide a foundational methodological framework for interface design in multi-user collaborative systems, given its high-level abstraction and applicability to diverse design processes.

## 3. Method Framework

**Figure 1:**
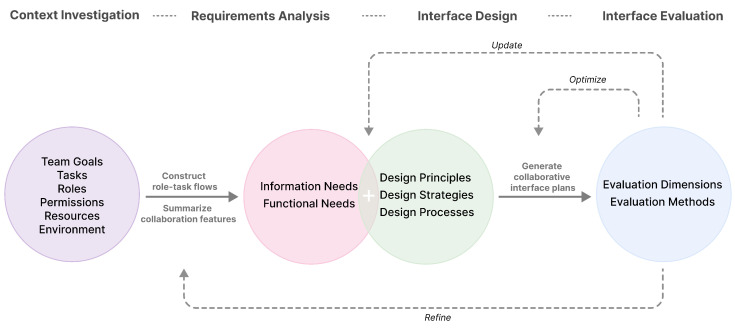
Method framework of role- and task-oriented multi-user collaborative interface design.

The construction methods for collaboration models effectively combine roles and tasks to build the collaborative process. User-centered design methods help extract user needs from the context of use, identify design opportunities, and enable continuous iteration and optimization. These two approaches offer a new perspective by extending single-user interface design methods to be applicable to multi-user collaborative interface design. Therefore, we will integrate these approaches to consider multi-user collaboration at various stages of the interface design process. We will particularly focus on the key factors of task processes and role relationships in collaboration, proposing a role- and task-oriented method for designing multi-user collaborative interfaces. Figure 1 illustrates the framework of this design method, divided into four key parts:

Part 1: Collaboration Context Investigation Understand the overall situation of the target collaborative group and analyze their typical collaborative characteristics. We suggest conducting the context investigation by defining the team’s collaboration goals, understanding the specific processes of collaborative tasks, outlining the main roles within the team, defining the responsibilities and permissions of each role, organizing various collaborative resources, and analyzing the collaborative environment.

Part 2: Collaboration Requirements Analysis Analyze the collected context information comprehensively to extract and identify informational and functional requirements in the collaboration process. Informational requirements emphasize the necessary information for roles to cooperate with each other and complete their tasks, while functional requirements focus on providing appropriate collaboration services to the team and its individual members. We propose constructing a clear and orderly role-task process to extract the collaborative information needs of different roles and analyzing the typical characteristics of groupware systems to identify collaborative functional requirements.

Part 3: Collaborative Interface Design Design specific collaborative interface plans based on the collaboration context and requirements. We propose an extended understanding of existing interface design principles, strategies, and processes, emphasizing the features of multi-user collaboration. Designers and developers can follow these principles and strategies to address the informational and functional needs identified in the collaboration process. By adhering to the collaborative interface design process, they can produce specific collaborative interface design plans.

Part 4: Collaborative Interface Evaluation Assess and optimize the interface design plans to ensure they effectively support multi-user collaboration. We recommend assessing and optimizing the interface design plans to ensure they effectively support multi-user collaboration. We recommend selecting appropriate evaluation methods from the dimensions of collaboration performance and experience to evaluate and optimize the collaborative interface design to achieve the desired outcomes.

Furthermore, the proposed framework incorporates multiple pathways for updates, optimization, and refinements, designed to support iterative cycles throughout the entire design process. Designers and developers of collaborative systems can flexibly select and focus on key components of the framework, adapting them to the specific needs and circumstances of the team and project.

In the following subsections, we will sequentially explain each part of this method framework. Section 4 will discuss the key elements to focus on when conducting collaboration context investigations. Section 5 will detail the process of extracting collaboration requirements. Section 6 and Section 7 will, respectively, cover the design and evaluation of collaborative interfaces.

## 4. Collaboration Context Investigation

**Figure 2:**
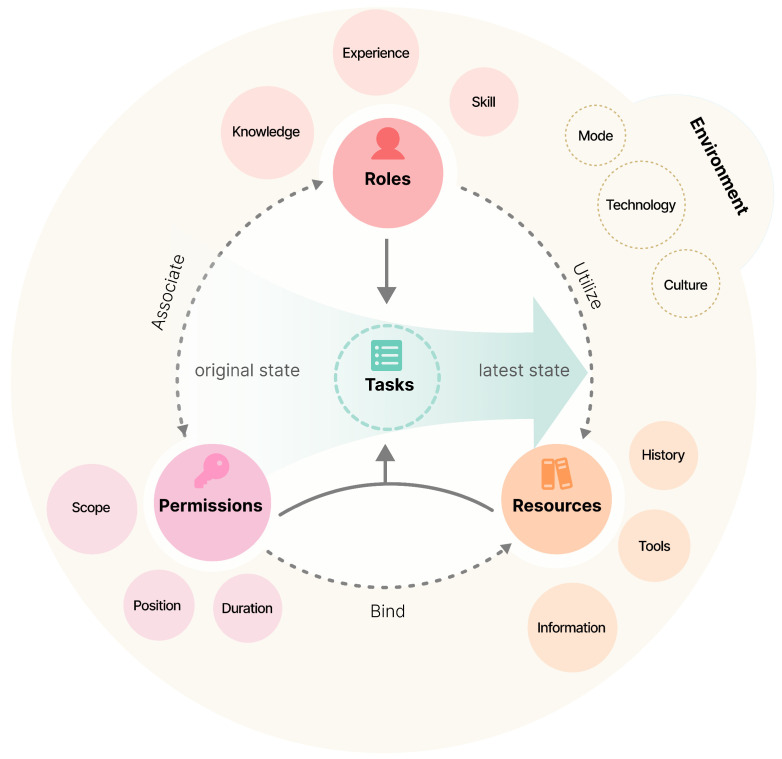
Key elements and relationships in collaboration context investigation.

The collaboration context can be investigated and analyzed from main five aspects: tasks, roles, permissions, resources, and environment. Tasks define the work the team needs to complete. Roles clarify the responsibilities of each member. Permissions ensure the allocation and use of resources. Resources include various types needed in the collaboration process, such as physical, informational, technical, and human resources. The environment encompasses collaboration modes and technologies as well as the physical and social contexts. Among these, tasks and roles are the core elements. The collaboration process can be viewed as each role utilizing their permissions to operate and change the state of the tasks or parts of tasks they are responsible for, as illustrated in Figure 2.

### 4.1. Collaboration Tasks

Collaboration tasks can be defined as a series of collaborative activities undertaken by people to achieve a common overall goal. Understanding collaboration tasks involves analyzing task relationships and decomposing task processes.

**Figure 3:**
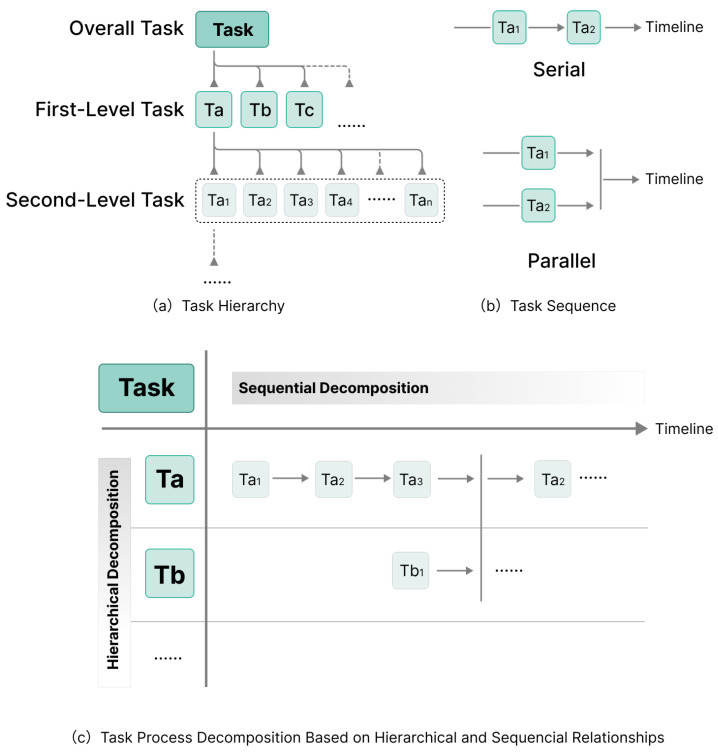
Task relationships and task process decomposition.

1. Task Relationship Analysis. Similar to individual tasks, collaboration tasks can be broken down hierarchically and sequentially to clearly understand the dependencies and subordination between tasks and their subtasks.
  - Task Hierarchy: The hierarchies of collaboration tasks can be decomposed by constructing Task Structure Trees [60,61]. In this structure, a general task can be broken down into multiple subtasks, each of which can be further divided into more specific subtasks, forming a tree-like hierarchy, as shown in Figure 3a. For example, in a building renovation project, the general task can be divided into major tasks such as preliminary preparation, design, construction, and acceptance. The construction task can be further divided into subtasks such as demolition, foundation work, main construction, and detailed decoration. Each subtask may include specific steps, such as floor paving, wall painting, and ceiling installation in the main construction. This hierarchical task decomposition reflects the process from general to specific, with each layer representing more detailed implementation steps of the previous layer, and the depth of the hierarchy indicating the level of detail in the task decomposition.
  - Task Sequence: The sequences of collaboration tasks primarily involve the order of task execution. In project management, tasks can be categorized into four types: Finish-to-Start (FS), Start-to-Start (SS), Finish-to-Finish (FF), and Start-to-Finish (SF) [62,63]. Although real-world scenarios may be more complex, they can generally be explained using two fundamental logical relationships: serial and parallel.
    - Serial Relationship: It defines the order of task execution, suitable for interdependent tasks where the execution of one task depends on the completion of another, forming a clear linear process, as shown in Figure 3b. For example, in construction, foundation work must be completed before the main structure is built.
    - Parallel Relationship: It allows multiple tasks to be performed simultaneously, suitable for independent tasks with no direct dependencies, as shown in Figure 3b. For instance, once the main structure of a building is completed, interior decoration and exterior landscaping can be designed simultaneously because they work in different areas and are independent of each other.
2. Task Process Decomposition. Combining hierarchical and sequential relationships for task process analysis provides a comprehensive understanding and management of tasks. As shown in Figure 3c, the horizontal axis displays the sequential relationships of tasks, specifying the particular time points or periods within which each task needs to be executed. The vertical axis represents tasks at the same hierarchical level in different sequences, indicating their specific levels within the project structure. This dual-dimensional analysis makes the task process clearer, aiding in dynamically adjusting project plans.

### 4.2. Collaboration Roles

Roles are a key method for directing design and development teams towards the user experience [64]. Collaboration roles are defined as empirically derived descriptions of hypothetical groups of individuals, characterized by specific qualities, goals, and needs, which are realized through their collaborative interactions [65]. Role definitions and their dependencies are specific interactional properties of collaboration.

**Figure 4:**
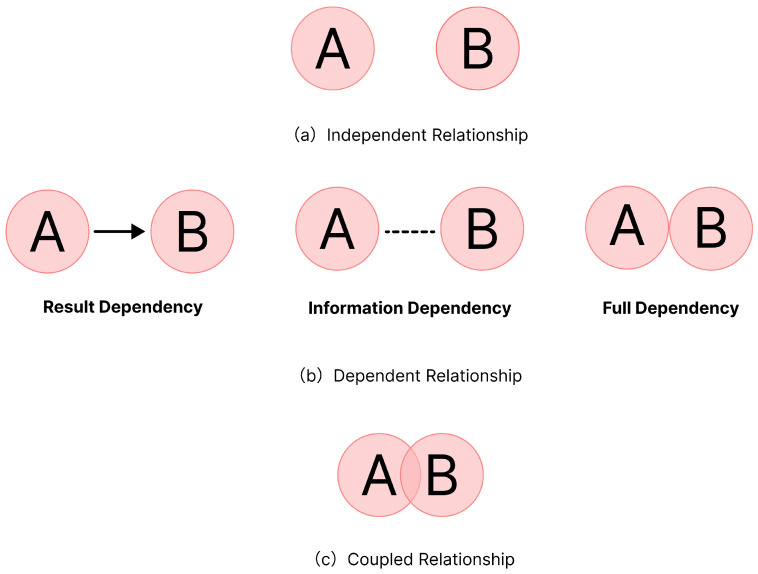
Role relationships.

1. Role Classification Setup. The classification of roles can be based on task characteristics, identifying the knowledge, skills, and experience needed to complete the tasks, and then creating a list of roles with specific responsibilities defined for each. For example, in a building renovation project, the project manager is responsible for overall planning and coordination, the designer develops the design plan, the construction engineer creates the construction drawings, the construction foreman leads the construction team, and electricians and carpenters are responsible for electrical systems and woodwork installation, respectively. These roles ensure the smooth progress of the construction project through close collaboration and effective communication.
2. Role Relationship Analysis. After defining the roles, it is essential to understand the relationships between them. Role relationships can be categorized into three main types based on the specific task requirements and the level of interdependence: independent relationships, dependent relationships, and coupled relationships.
  - Independent Relationship: This refers to each role performing their tasks independently without needing to rely on other roles. As shown in Figure 4a, Role A and Role B perform their own tasks independently. For example, in painting multiple rooms, each room can be painted by different workers independently. The painters have an independent collaboration relationship, focusing solely on their assigned rooms.
  - Dependent Relationship: This refers to one role completing its tasks depending on the support or output of another role. If Role A relies on the support provided by Role B to execute its task, Role B has a dependent collaboration relationship with Role A. Dependent collaboration can be further categorized based on the specific dependency:
    - Result Dependency: Role B’s task execution depends on the outcome of Role A’s task. For example, before painting a room, wall cleaning and repair must be completed, making the painter’s task dependent on the cleaning and repair workers’ results.
    - Information Dependency: Role B’s task execution relies on the information provided by Role A. For example, a painter needs information from the designer about paint colors and types but does not require the designer’s actual execution results.
    - Full Dependency: Role B’s task execution depends on both the information and the outcome provided by Role A. For example, for specific decorative effects, a painter may need the designer to specify paint colors and outline areas on the wall, making the task dependent on both the designer’s information and results.
  - Coupled Relationship: This reflects a deeper interdependence between roles, where each role’s task execution is closely linked and requires constant communication and coordination. As shown in Figure 4c, Role B’s task execution depends on Role A’s support, and vice versa. For example, in room renovation, electricians and HVAC (Heating, Ventilation, and Air Conditioning) technicians have a coupled collaboration relationship. Electricians need to lay wiring and install outlets, while HVAC technicians need to install heating and air conditioning ducts. They need to communicate continuously to ensure that wiring and duct installation are properly coordinated within the same space, ensuring the safety and functionality of the systems.

### 4.3. Collaboration Permissions

Different roles have different access permissions in the collaborative systems. The Role-Based Access Control (RBAC) model associates permissions with roles and assigns users to appropriate roles to grant them the corresponding permissions [66,67,68]. Permissions determine the level of access users have to system resources, including access scope, location and duration. For example, in a renovation project, the project manager is responsible for overall coordination and managing the project schedule. Only the project manager can access and modify project files and plans, assign tasks, adjust team members’ responsibilities, and approve procurement and budget changes.

User interfaces often utilize permission-driven designs, displaying or hiding information or functionalities based on the user’s permission level, which can be highly beneficial in collaborative systems. Clear permission settings not only improve the efficiency of executing collaborative tasks but also prevent abuse of permissions and security issues when multiple users have conflicting, complex, and dynamic needs for resources. Additionally, since permissions are associated with roles rather than individual users, they can be flexibly managed to handle frequent changes in individual user assignments.

### 4.4. Collaboration Resources

Collaboration resources refer to the various supportive elements needed to complete specific tasks in the team collaboration process, aiding roles in altering the state of tasks. These resources can include human resources, physical resources, information, tools, and more, providing the necessary conditions for team members to complete their tasks. Mingjun (2007) more specifically classified collaboration resources into goal resources, state resources, boundary information resources, interaction tool resources, feedback resources, historical resources, and knowledge resources [69]. Effective management of collaboration resources can enhance the productivity, efficiency, and quality of cooperation within the team.

### 4.5. Collaboration Environment

The collaboration environment can be understood as the physical and virtual working conditions and the support systems within which a team operates while executing tasks or projects. Currently, collaboration heavily relies on information exchange between people, facilitated by information and communication technology. Therefore, the collaboration environment can be emphasized as a shared information space. This space includes data objects generated and used during collaboration, the specific actions of team users, and the impact of these actions on the state of data objects. When analyzing the collaboration environment, we need to particularly consider collaboration modes and the supporting technologies, as they also determine the characteristics of the collaboration interface.

Collaboration modes are typically categorized into synchronous, asynchronous, co-located, and distributed [22]. In synchronous collaboration, team members exchange information in real-time, allowing simultaneous editing of shared objects, with changes instantly visible to other members. Conversely, asynchronous collaboration permits time-delayed information exchange between members. Co-located collaboration involves team members being in the same physical space, enabling face-to-face communication and cooperation. In contrast, distributed collaboration involves team members spread across different geographical locations, relying on digital communication tools for interaction and collaboration. Different collaboration modes are suitable for different collaborative needs. Teams can choose the most appropriate collaboration method based on specific project and team characteristics, thereby enhancing collaboration efficiency and project success rates.

Different collaboration modes require different technologies to support specific communication and coordination needs. Synchronous collaboration relies more on real-time communication tools and interactive devices, while asynchronous collaboration depends more on project management platforms and document management systems. Co-located collaboration demands more from the physical space and equipment, such as shared meeting rooms and large or multi-screen displays for face-to-face discussions and collaborative operations. Distributed collaboration, on the other hand, relies more on efficient communication and collaboration software to ensure that geographically dispersed team members can effectively communicate. Choosing the appropriate collaboration technology can improve team efficiency, enhance communication, and improve the collaboration experience.

Additionally, team culture, social norms, and interpersonal relationships are also important aspects of the collaboration environment. Overall, the collaboration environment is not only a collection point for information but also the foundation for maintaining the continuity and integrity of collaborative activities.

## 5. Collaboration Requirements Analysis

The process of requirement analysis and specification is one of the most critical parts of any system development [70]. In collaborative systems, information serves as the foundation for users to complete tasks, while functions are the tools and services provided by the system to help users obtain, share information, and perform tasks. In this section, we conduct information-level requirement analysis centered on collaboration task processes and role relationships and perform function-level requirement analysis based on the characteristics of groupware. This helps to clarify the information users need during collaboration and identify the specific functional modules that support these information acquisition and task execution needs.

### 5.1. Information Requirement

The multi-user collaboration process involves members sharing, transmitting, understanding, and providing feedback on information through various channels and methods. A key characteristic of cooperative systems is the dissemination of activity information among group members. Information Requirements Determination (IRD) is the most critical stage in information system development [71]. Therefore, it is essential to analyze when and in what form different roles use information during collaboration. This can be achieved by constructing task flows and information flows that incorporate role relationships.

**Figure 5:**
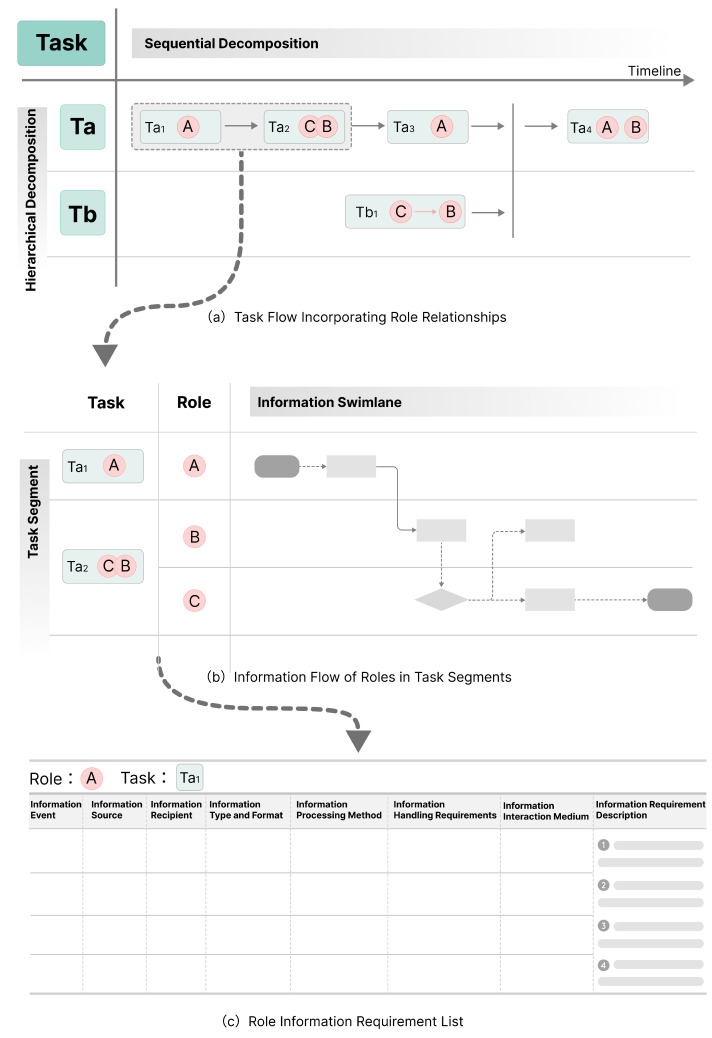
Process of extracting role information requirements.

1. Task Flow Construction. By incorporating roles into task flow diagrams [72,73], as shown in Figure 5a, the specific responsibilities and collaboration relationships of different roles within the team can be clearly defined. First, based on the timeline, list the main tasks and time stages of the project to reflect the sequential relationships between tasks. Then, indicate the role responsible for each task, showing the correspondence between roles and tasks and the collaborative relationships among the roles executing those tasks.
2. Information Flow Construction. Extract typical task flow segments from the task flow incorporating role relationships and further construct the specific information flow for roles in subtasks, as shown in Figure 5b. Create a swimlane [74,75,76] for each role, demonstrating the process of sending and receiving information during different task stages. Arrows should represent the direction of information flow, and symbols or brief text should describe the information content. To ensure clarity and accuracy, the elements of the swimlane need to be standardized, including starting information events, process information events, decision information events, task switch information flows, information flows, and annotation texts [77].
3. Role Information Requirements Extraction. Only by thoroughly understanding the roles and their related tasks can one truly comprehend the reasons, methods, and timing of information searches [78,79]. The information swimlane can be used to further organize the list of information requirements for each role, clarifying information sources, processing methods, and transmission forms to obtain the information needs of each role.
  - Analyzing Information Needs: Analyze the information swimlane to determine the types of information each role requires at different task stages. This includes receiving (sources and types of information), processing (handling and analyzing received information), and outputting information (recipients, form, and frequency).
  - Detailing Information Needs: Create a detailed list of information needs for each role, specifying requirements for specific tasks. This includes information events (task events in the information interaction process), information sources and recipients (origins and receivers of the information), types and formats (documents, reports, charts), processing methods (summarizing, analyzing, reporting), processing requirements (completion, format, time, confidentiality), and exchange mediums (devices, sensors, terminals). Describe these needs to clarify the role’s information requirements for the task (Figure 5c).
  - Defining Information Needs: Needs are subjective and can be inferred from behavior or self-reporting [80]. Discuss the summarized list with the roles to collect feedback. Adjust and optimize based on the feedback to ensure accuracy. Methods include closed-ended questions [81], open-ended questions [81], brainstorming [82,83], guided brainstorming [84], and group consensus [85,86]. Choose methods based on the collaboration scenario and tasks.

### 5.2. Functional Requirement

**Figure 6:**
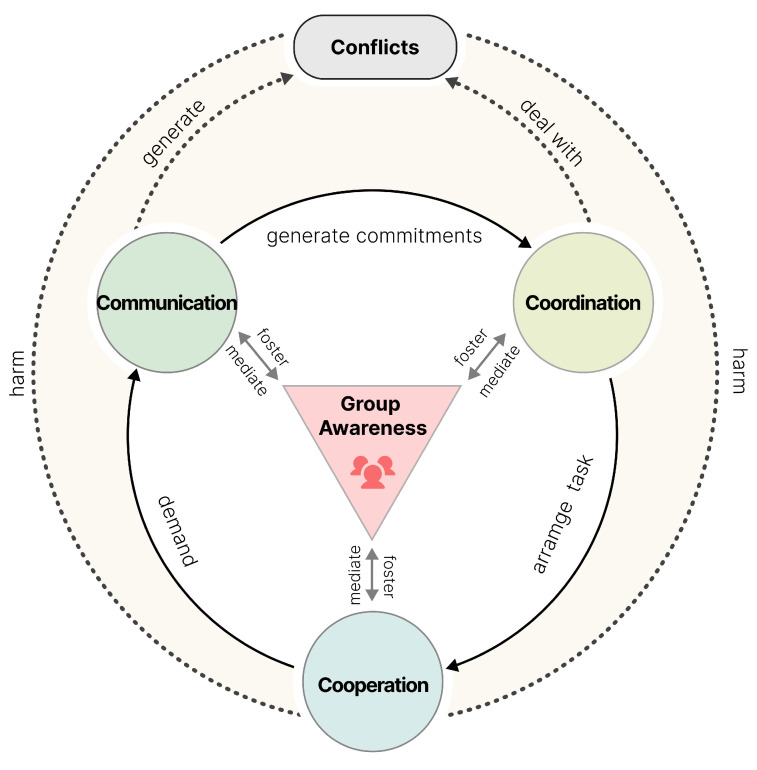
The 3C collaboration model [88].

The infrastructure of groupware includes three important features: communication (pushing or pulling information within the organization), collaboration (using shared information and establishing shared understanding), and coordination (concurrency control and latecomer support) [87]. The relationship between these features [88] is shown in Figure 6. Among these, communication is the core requirement. Effective communication ensures clear and timely information transmission, promoting collaboration awareness, team members’ recognition and understanding of the collaborative environment and each other’s actions. Communication within the team not only involves information exchange but also the creation of commitments and expectations, such as agreeing on task completion or adhering to specific deadlines. Coordination oversees these commitments by ensuring smooth task allocation, role clarity, progress tracking, and appropriate resource distribution within the team.

Groupware applications have specific functionalities that are considered to enhance the quality of the user interface [89]. Conversely, the user interface is a key factor affecting the acceptance of groupware applications because it facilitates communication and collaboration among users working on collaborative tasks [90]. When designing and developing a complex collaborative system used simultaneously by different users, methods and techniques related to user interface development should consider the specific features of groupware [90]. By analyzing these features, we can understand the functional needs of users in the collaborative system.

1. Awareness. Dourish and Bellotti (1992) defined collaboration awareness as understanding others’ actions and controlling one’s own to avoid conflicts, relating to the group’s overall behavior [91]. Collaboration awareness includes role awareness (understanding team members’ roles, responsibilities, permissions, resource management, competence levels, and task assignments), action awareness (monitoring activities to understand progress and predict future actions), time awareness (shared understanding of task timing and deadlines), and space awareness (knowledge of members’ physical or virtual positions, the collaborative environment, and surrounding resources).
2. Communication. Communication involves transmitting and receiving information between team members, divided into explicit and implicit communication [92]. Explicit communication is intentional and planned, including verbal (face-to-face, calls, video conferences), written (emails, instant messages, reports, memos), and gestural (body language) exchanges. Implicit communication is unintentional, relying on environmental cues, behavior patterns, and work traces to gather information, such as unconsciously perceiving activities, status, and progress in a shared workspace, maintaining a mutual understanding of team dynamics.
3. Coordination. Coordination involves managing activities, resources, and information among team members to achieve common goals [93,94]. It includes shared access and transfer. Shared access manages team members’ use of resources, including acquisition, reservation, and retention. Transfer involves moving resources, tasks, and information among members, including task allocation, role changes, and providing necessary support.
4. Conflict. Conflict arises among team members due to differences or inconsistencies in goals, methods, or resource allocation. Conflicts usually stem from differences in awareness, insufficient communication, or failed coordination.

Based on the above analysis, we derived the macro-functional requirements for collaborative interface design, emphasizing that the interface should promote mutual awareness, support efficient communication, clarify coordination responsibilities, and effectively manage conflicts.

## 6. Collaborative Interface Design

Adhering to reasonable and effective design principles, strategies, and processes can directly enhance the quality of the interface design. Although most design principles are aimed at single-user interfaces, the interface between an individual user and the computer still exists in collaborative systems, making some guidelines for single-user interfaces still applicable. By further integrating considerations of collaboration requirements and features, we can achieve better collaborative interface design.

### 6.1. Collaborative Interface Design Principles

There are many interface design principles, such as Johnson’s nine principles [95], Shneiderman’s eight golden rules [96], and Gestalt principles [97]. Although there is no complete consensus on the primary design principles, most of them share some core ideas. Hewitt and Gilbert summarized five universal interface design principles and discussed their application to the issues and challenges in groupware systems [98]. Based on our understanding, we further expand these principles for collaborative systems.

1. Maintain Consistency. In collaborative systems, users can access both shared and personal workspaces, which should maintain consistency in display and operation. Display consistency involves visual perception, meaning all collaborators should have the same visual experience when using the system. For instance, the activities and identities of different users should have uniform visual indicators in the interface, making it easier to identify team roles and distinguish between individual users. The creation, assignment, and completion processes of collaborative tasks should have consistent visual representations across all user interfaces. Operational consistency means all users should use the same interaction methods and tools for similar tasks, ensuring that collaborators understand each other’s actions. For example, the affordances of collaborative function modules and the methods of sharing collaborative information should be consistent.
2. Provide Immediate Feedback. In collaborative systems, feedback comes from multiple channels, including system feedback and interaction feedback from other members. The system should immediately update and feedback on collaborators’ activity statuses and their effects on collaborative tasks. It should also allow active reminders and sharing from other collaborators. Feedback should be diverse, allowing various methods and enabling users to customize their preferred feedback formats. Additionally, social feedback mechanisms should be encouraged, displaying interactions like likes, comments, and replies among users to enhance team interaction and cohesion.
3. Use the User’s Model. In collaborative systems, the user model should include individual behaviors and needs and extend to the group level, reflecting dynamic team interactions. Building and using collaborative models can help all users understand how the system supports collaboration, such as interaction modes and communication paths. The user model should also be dynamically adjustable and continuously optimized based on user behavior data and feedback during the collaboration process, ensuring it always reflects the latest collaboration needs and behavior patterns.
4. User-Centered Control. In collaboration systems, users must have clear visibility into the current state of other roles and tasks, their previous states, and potential future states to maintain control. This involves providing real-time updates, shared dashboards, and activity feeds that display the latest actions and changes made by collaborators. Access to a history of actions and changes through version control systems and change logs helps users understand the project’s evolution. Predictive analytics and task dependency maps can assist users in planning their next steps.
5. Use Concrete Metaphors. In collaborative systems, real-world collaboration scenarios and experiences should be used to introduce new metaphors, such as meeting room metaphors, task board metaphors, social network metaphors, and transaction and agreement metaphors. These metaphors help group users leverage previous collaborative experiences, whether from the ’real world’ or other applications. This approach facilitates faster learning and enables users to make appropriate inferences about the interface based on their existing knowledge, helping them understand the collaborative system and adapt to the collaborative environment.

### 6.2. Collaborative Interface Design Strategies

Based on the analysis in Section 5.2, we further summarize collaborative interface design strategies from four dimensions: enhancing collaborative awareness, promoting effective communication, clarifying coordination mechanisms, and avoiding perceptual conflicts.

1. Enhancing Collaborative Awareness. In a collaborative environment, the complexity is higher, and an individual’s perception often fails to meet task demands. Collective awareness can enhance the overall team’s situation awareness [99]. Design can focus on improving role information awareness, action awareness, time awareness, and space awareness.
  - Role Awareness: Clearly define each team member’s roles and responsibilities and ensure that their relevant information (who they are, their capabilities, permissions, and resources) is transparent and accessible to everyone. Methods to enhance role information awareness include shared cursors [100] and role lists. Shared cursors allow each member to have their own cursor, differentiated by color, user name, user picture, or personalized cursors, enhancing role awareness. With the development of sensing and operation technologies, shared cursors extend to shared gesture prompts and shared gaze visualization [101,102]. Role lists present role identities, online status, and role relationships in a list format, using names, pictures, and auxiliary text to help members understand the identities and status of each role in the current collaborative environment.
  - Action Awareness: Understand the actions and progress of other collaborators, ensuring transparent workflows and decision-making processes. Methods to enhance action awareness include shared views [103,104] and multi-user scrollbars [105]. Shared views allow collaborators to share first-person perspectives, enhancing understanding of each other’s actions. Multi-user scrollbars mark all collaborative content of a user with colors, names, and labels on the scrollbar, allowing other collaborators to locate specific content directly.
  - Time Awareness: Establish a common timeline to manage and synchronize team members’ task schedules. Shared calendars are a way to enhance time awareness by sharing time plans and rhythms [106,107]. Through shared calendars, teams can set up meetings, important deadlines, and reminders, making all members aware of their schedules and those of others.
  - Space Awareness: Use digital tools to map and share the positions of other members in physical or virtual workspaces. One method is using mini-maps or radar displays [108,109], providing a miniature view of the entire shared workspace with different colors or symbols marking each member’s position.
  The use of these methods needs to match the appropriate usage scenarios. In cases of complex relationships, high real-time requirements, and high-security needs, adopting comprehensive situational awareness mechanisms and strategies can better handle complex collaborative environments, improving team efficiency and experience.
2. Promoting Effective Communication. Effective communication ensures timely transmission and understanding of information, enhancing awareness and preventing conflicts, thereby improving collaboration efficiency among team members. Strengthening explicit communication channels and creating implicit communication environments can promote effective communication. Design can focus on intuitive interaction, auxiliary communication, information sharing, visual expression, and historical information review.
  - Intuitive Interaction: In co-located collaboration, verbal communication, gestures, and eye contact are the most direct ways to express collaborative intentions. In distributed scenarios, team members can use remote communication tools like video conferencing systems and instant messaging platforms. Additionally, digital tools can simulate co-located interactions, such as using VR technology and virtual avatars to place everyone in the same virtual scene [110].
  - Auxiliary Communication: In co-located environments, collaboration tools like whiteboards, desktop interaction devices [111], or interactive walls [112] can enhance communication. Additionally, multimodal sensing technology combined with collaboration devices can improve communication, such as shared pointing visualization, where the extended line from the speaker’s finger intersects with the shared screen, focusing other members on the current shared content [113]. In distributed environments, increasing communication feedback mechanisms can help collaborators understand each other’s intentions, for example, by allowing remote partners to use remote gestures in video systems to complete collaborative physical tasks [114].
  - Information Sharing: Provide a centralized information platform for team members to easily share and access key information like documents, plans, and reports. Consider role-specific needs to achieve personalized information resource sharing.
  - Visual Expression: Using visual representations based on graphics, images, and numerical elements can facilitate the understanding of abstract or complex concepts, effectively reducing cognitive load and errors among team members. Visual representations can share team activities, collaboration mechanisms, role responsibilities, task progress, and data metrics, providing a clear understanding of complex information.
  - Historical Information Review: Allow collaborators to review and analyze the communication and task content history, tracking each other’s participation [115]. This includes current task progress, past milestone completion, deliverables and version records, special case records, and communication processes, ensuring anomalies can be traced and responsible parties contacted immediately.
3. Clarifying Coordination Mechanisms. Coordination mechanisms manage dependencies and organize processes, entities, and arrangements to enhance group performance [116]. In multi-user collaborative environments, clarifying coordination mechanisms aims to optimize collaboration processes, reduce conflicts, and improve team efficiency through clear rule-setting.
  - Role/Permission Differentiation: Collaboration content has privacy and information security requirements, with permissions to view, edit, or participate restricted to relevant personnel. Roles can be classified by organizational hierarchy, function, or project/task ownership. Permissions can be set in automatic or manual modes. Automatic settings bind roles and permissions based on preset rules executed directly according to task allocation and flow, while manual settings suit flexible projects and tasks where roles are not fixed.
  - Plans and Rules: Establish plans to build consensus, such as project management plans, design standards, or delivery schedules, to alleviate coordination conflicts by enhancing transparency and predictability. Establishing rules ensures resource availability, detailing how, when, and to whom resources are allocated to promote project coordination.
  - Monitoring: Monitoring can be automatic or conducted by high-permission roles overseeing the team’s overall activities, intervening when necessary, and ensuring smooth task progress.
  - Space Layout and Personal Territories: Shared digital workspaces increase visibility and awareness of each other’s work. Collaborators tend to divide their work areas into personal, group, and storage areas [117]. Pay attention to the representation and transfer mechanisms of system resources in different spaces.
  - Flexible Adjustment and Redistribution: Adjust and redistribute tasks flexibly when task requirements or role permissions change, maintaining team adaptability and responsiveness while reducing conflicts or failures due to outdated or impractical task arrangements.
4. Avoiding Perceptual Conflicts. In collaborative environments, concurrent access to shared objects by multiple users is inevitable, leading to conflicts. The solution involves incorporating coordination strategies to manage all operations sent to the server, ensuring relevant collaboration roles receive timely and consistent collaboration information.
  - Role Permission Awareness: Define and communicate operation rights and responsibilities when establishing roles, ensuring all roles understand their own and others’ permissions, preventing neglect and unauthorized actions.
  - Operation Feedback and Locking: After a role performs an operation on an object, feedback should be sent to other relevant collaborators, indicating events initiated by shared object operations. Operation locking should protect current operations and prevent interference, reporting to other relevant collaborators about who locked the shared object and why, and facilitating reasonable negotiations based on the current collaboration state.

### 6.3. Collaborative Interface Design Process

**Figure 7:**
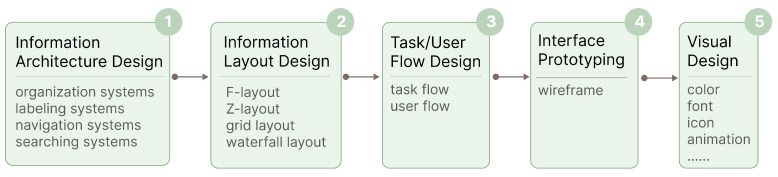
Collaborative interface design process.

The design process for collaborative interfaces mirrors that of single-user interfaces and can be broken down into five key parts: information architecture design, interface layout design, task/user flow design, interface prototyping, and visual design, as shown in Figure 7.

1. Information Architecture Design. An interface comprises numerous information elements organized according to specific rules. These elements, the smallest interactive units, include text, images, charts, and buttons. Information architecture design involves logically organizing and expressing these elements, making it easier for users to access and understand the needed information. Information architecture consists of four components: organization systems (divide and organize information), labeling systems (present information), navigation systems (how users browse or move through information), and searching systems (how users look up information) [118]. Brown (2010) proposed eight principles for information architecture [119]: objects, choices, disclosure, exemplars, front doors, multiple classification, focused navigation, and growth, which can guide the design process. For collaborative systems, it is crucial to consider when, where, and how different collaborative information should be presented to the target roles to help them access the necessary information.
2. Information Layout Design. The goal of information layout design is to group and organize large, complex information to facilitate efficient information search and task execution [120]. Although there are no explicit guidelines for task-based or ecosystem-based interface design [121], four mainstream information layout methods are commonly used: F-layout (following the user’s left-to-right, top-to-bottom reading habits), Z-layout (minimizing obstacles in the browsing path, suitable for pages with less text content), grid layout (structuring information into multiple modules or blocks using a grid), and waterfall layout (arranging images or content blocks of varying heights closely together for a natural flow visual effect). In collaborative interface layout design, the overall layout should align with different roles’ understanding of their collaborative relationships and respective responsibilities, ensuring that each role can intuitively comprehend the logical relationships between information and functions while performing tasks. The placement of information and functional blocks should be based on role responsibilities, task importance, dependencies, and execution order. Information related to high-priority and high-frequency tasks should be placed in prominent positions on the interface to ensure easy access and use. The layout of functional blocks should reflect the inherent attributes of tasks and meet operational logic, ensuring users can easily navigate and use the interface. For example, information and functions related to closely connected tasks or roles should be positioned together to minimize the time and complexity of switching between different interfaces.
3. Task/User Flow Design. Task flow design should clearly display each step a role takes to complete a specific task, while user flow design needs to cover the overall journey of a role within the system, encompassing multiple task flows and involving user decision points and system responses. Special attention should be given to communication and interaction between roles and task handovers. Clarify the interaction points of each role at different task stages to ensure smooth information and task flows. Design clear task handover mechanisms to facilitate the seamless transfer of tasks from one role to another.
4. Interface Prototyping. Translate abstract information structures and functional modules into specific product interaction solutions, typically expressed through wireframes that outline the interface layout and interaction modes. In collaborative interface prototyping, collaborative interface design strategies should be implemented into concrete interface elements and interaction details.
5. Visual Design. Incorporate aesthetic elements to create high-fidelity design prototypes. First, determine the overall design style based on the team’s style and goals, and extend this to a matching color scheme. Additionally, consider details such as font selection, icon design, and the application of animations, ensuring consistency and coherence among all elements. Finally, combine the overall style with interaction details to present a visually appealing and practical interface.

## 7. Collaborative Interface Evaluation

To identify usability issues in the designed interface, the most common method is user testing. User testing can further reveal user needs, preferences, encountered problems, and potential improvement points. Below, we discuss the evaluation dimensions and specific evaluation methods for collaborative interfaces.

### 7.1. Evaluation Dimensions

When testing and evaluating the interface of multi-user collaboration systems, we can consider two dimensions: collaborative performance and collaborative experience.

1. Collaborative Performance. This directly impacts the speed and quality of task completion by the team and is a key aspect of evaluating the quality of multi-user collaboration. Collaborative performance can be assessed from three perspectives: task completion time, accuracy, and team cognition.
  - Task Completion Time: This is a direct indicator of the time required to complete a specific collaborative task, reflecting the operational efficiency of collaborators. Compared to the task completion time of a single user, the time for collaborative tasks can be divided into two parts: the time taken by each role to complete their respective tasks and the time spent on task flow and handover. By comparing the time required for multiple users to complete a task under different interfaces, we can directly evaluate the impact of the collaborative interface on collaboration efficiency.
  - Task Accuracy: This refers to the success rate of team members completing tasks using the collaborative system. This metric is particularly important in tasks requiring high precision and strong knowledge coordination. By setting challenging tasks and recording the number of errors and corrections, we can assess the team’s ability to avoid mistakes and errors using the collaborative interface.
  - Team Cognition: This refers to the team members’ understanding of the team task, including how the task is organized, represented, and allocated [122]. Many studies have confirmed a strong positive correlation between team cognition and team collaboration performance [123]. Team cognition arises from interactions among team members [124], and high-quality task-oriented communication can promote the development of team cognition, while poor communication may inhibit it [125]. Team interviews and surveys can be used to evaluate changes in team cognition during collaboration.
2. Collaborative Experience. In collaborative scenarios, the user experience of multi-role collaborative interfaces refers to the experience gained by different roles using the system together. This experience includes not only each individual user’s experience but also the interaction experience between users. Collaborative experience can be understood as the sum of the user experiences of all roles participating in the task. We can use the User Experience Questionnaire (UEQ) to measure this. The questionnaire covers two comprehensive impressions and six dimensions of user experience [126,127,128,129]: efficiency, clarity, reliability (pragmatic quality) and attractiveness, stimulation, novelty (hedonic quality).

### 7.2. Evaluation Methods

Methods for multi-user testing should focus on supporting group collaboration, facilitating social interactions, and ensuring usability for systems serving multiple users. Table 1 lists several common multi-user testing methods.

In multi-user testing and evaluation, it can be challenging to directly invite real users to participate. Therefore, during the design evaluation phase, alternative methods can be used to simulate the user testing environment and collect user feedback. Common methods include the role-playing method [137,138,139] and Wizard of Oz testing [140,141]. In the role-playing method, designers create detailed personas based on preliminary research and user survey data, with each persona representing a specific user group. By simulating interactions between these personas and the product or service in specific scenarios, designers can predict and evaluate potential issues in the collaboration process [142]. Wizard of Oz testing is suitable for early development stages. When some system or product functions are not yet implemented but need to be tested for user acceptance or operational flow, designers can simulate parts or all functions manually, making users believe they are interacting with a real system. In multi-user collaboration scenarios, several users can be invited to experience the system simultaneously, observing how they collaborate to solve problems and complete tasks together.

## 8. Outlooks and Limitations

### 8.1. Outlooks

As technologies rapidly evolve, collaborative contexts are expanding, and new challenges continually arise. In particular, the rapid development of advanced technologies such as artificial intelligence (AI) has not only significantly broadened collaborative application scenarios but has also made the roles and tasks within collaborative systems increasingly complex, diverse, and difficult to manage.

In emerging collaborative systems, collaborators are no longer limited to humans but now include various virtual intelligent agents with different levels of intelligence and functional characteristics [143], as well as physical robots [144,145]. The introduction of these AI collaborators is reshaping the inherent cooperation relationship and team structures among humans. We are transitioning toward a new paradigm of human–machine teams [146], where AI collaborators are becoming an integral part of the collaborative team, playing crucial roles in performing complex tasks alongside humans. Beyond traditional supportive roles, such as aligning with users’ habits, preferences, and skill levels [147,148], and assisting with workflow optimization [149,150]. AI collaborators are increasingly evolving into higher-level strategic partners. In these roles, they can act as creative guides or risk monitors in complex scenarios. By leveraging contextual understanding and intent interpretation, AI can inspire creativity within team members [151,152]. Furthermore, through large-scale data analysis and predictive modeling, AI collaborators can identify potential risks, provide early warnings, and improve the accuracy of team decision-making [153].

However, despite the significant benefits that AI collaborators bring to collaborative systems, they also introduce notable challenges. First, AI’s decision-making strategies are often based on complex algorithms rather than explicit logical reasoning, resulting in a “black-box” nature that makes it difficult for human team members to understand the rationale behind AI behaviors [154,155]. This lack of transparency can foster distrust or resistance among team members, undermining the trust essential for effective collaboration. Second, while AI’s computational efficiency and standardized interventions can greatly enhance collaborative performance, they may also lead human participants to feel “suppressed” or marginalized within the team [156]. Over-reliance on AI recommendations may reduce human team members’ engagement and initiative, ultimately weakening intellectual diversity and decision-making capacity. More critically, AI decision-making processes, which are heavily reliant on training data, may inherit or amplify biases present in the data [157,158]. Such biases could manifest as favoritism or prejudice in resource allocation and task coordination [159,160], potentially exacerbating power imbalances within teams and creating new collaboration conflicts, thereby limiting overall team effectiveness.

Collaborative tasks are also becoming increasingly diverse, characterized by cross-disciplinary, cross-domain, cross-cultural, and cross-temporal collaboration. These complexities demand greater reliance on advanced technologies and data-driven solutions for communication, coordination, and execution. On the one hand, the interoperability of multiple technological modules remains a persistent challenge [161]. Without proper integration, collaboration systems risk developing information silos or experiencing disruptions that can hinder team efficiency. Additionally, the introduction of new collaborative tools often requires significant time and effort for team members to learn and adapt, increasing cognitive load and psychological stress, particularly for non-technical users who may face significant barriers to adoption. On the other hand, the successful execution of collaborative tasks increasingly relies on the real-time collection, integration, and analysis of multimodal data from both individual and team levels to enable dynamic task adjustments and resource optimization. However, achieving a balance between data sharing and protecting the privacy and security of collaborative data has become a critical design challenge for collaborative systems [154,162].

In summary, these developments introduce new demands for the design of future collaborative interfaces, as well as new requirements for design methodologies that guide practical applications. From a role design perspective, collaborative interfaces must clearly define the roles of AI collaborators and employ visualization techniques to dynamically present AI decision-making processes and operational logic, enhancing interpretability and fostering user trust. From a task design perspective, it is essential to optimize collaboration processes based on the complexity of tasks and the diversity of teams, ensuring efficient task distribution and resource coordination while maintaining system flexibility and adaptability.

### 8.2. Limitations

The design methods discussed in this study are based on current theories and practical solutions in collaborative interaction research, giving them a degree of universality. However, we acknowledge that validating its effectiveness through practical application remains a critical next step. Future research will focus on selecting representative collaborative groups and scenarios to conduct case studies, documenting the implementation process and evaluating its outcomes. Additionally, controlled experiments will be designed to collect user feedback and behavioral data, enabling comparisons of key collaborative performance metrics before and after the adoption of the proposed design method. These findings will serve as the basis for refining and optimizing the methodology, thereby enhancing its practical value and applicability.

Furthermore, it is essential to further investigate the scalability and customizability of the collaborative interface design method proposed in this study within discipline-specific collaborative environments. The systematic integration of domain-specific collaborative knowledge and professional expertise is expected to enhance the applicability and usability of this design methodology across diverse industries.

Overall, research on multi-user collaboration should emphasize the combination of practice and theory. Providing deeper theoretical insights to guide practical implementation and using practical experiences to enrich theoretical foundations are essential. Both aspects should progress in parallel to collectively promote the development of collaborative systems towards greater efficiency, flexibility, user-friendliness, and intelligence.

## 9. Conclusions

This paper presents a comprehensive approach to analyzing task processes and role relationships in multi-user collaborative scenarios and constructs an interface design method that emphasizes the interaction characteristics of multi-user collaborative systems. Based on collaborative model construction methods and user-centered design approaches, this method offers a thorough design framework and provides detailed design step guidance, covering context research, requirement analysis, interface design, and evaluation. The aim is to help create and optimize collaborative interface designs through standardized methods and detailed processes.

## Figures and Tables

**Table 1:** Common multi-user testing methods.

Testing Method	Description	Features	Application Scenarios
Parallel User Testing [130]	Invite multiple users to participate simultaneously in a controlled environment, allowing them to independently complete predefined tasks while observing and recording their behavior and feedback.	Can be conducted in a lab or controlled setting, effective in identifying design issues and improving user satisfaction.	Suitable for early stage product testing, quickly verifying design concepts and discovering issues in multi-user interactions.
A/B Testing [131]	Randomly assign users to different product versions to compare the impact of different designs or features on user behavior.	Useful for data analysis and statistical comparison, accurately measuring the effects of different designs.	Ideal for optimizing user interfaces and experiences, especially for comparing the impact of designs or features on behavior or increasing engagement.
Focus Groups [132]	A group of users, led by a moderator, discusses the product, providing insight into user attitudes, perceptions, and overall impressions.	Provides direct user feedback in a controlled setting, helping to understand user attitudes and emotional responses.	Useful for gathering early-stage user feedback on product concepts or functionalities, particularly for exploring user needs and refining products.
Guerrilla Testing [133]	A quick and cost-effective method where testers randomly approach users in public places for short tests.	Low cost, fast feedback, but results may not fully meet target criteria.	Useful for early-stage validation of concepts or interfaces, needing diverse feedback.
Diary Studies [134,135]	Require users to record their experiences with the product over time.	Collects long-term data on user experiences and behaviors.	Suitable for understanding long-term user behavior trends and forming habitual usage patterns.
Remote Testing [136]	Tracks user data remotely through software embedded in the product, such as click rates and page visit durations.	Provides broad, large-scale usage data without requiring direct user participation.	Useful for understanding actual usage scenarios and identifying user needs and pain points on a large scale.

## Data Availability

No new data were created or analyzed in this study.
